# Clinical characteristics, treatment, and treatment switch after molecular‐genetic classification in individuals with maturity‐onset diabetes of the young: Insights from the multicenter real‐world DPV registry

**DOI:** 10.1111/1753-0407.70028

**Published:** 2024-11-07

**Authors:** Stefanie Lanzinger, Katharina Laubner, Katharina Warncke, Julia K. Mader, Sebastian Kummer, Claudia Boettcher, Torben Biester, Angela Galler, Daniela Klose, Reinhard W. Holl

**Affiliations:** ^1^ Institute of Epidemiology and Medical Biometry, CAQM Ulm University Ulm Germany; ^2^ Munich‐Neuherberg German Center for Diabetes Research (DZD) Munich Germany; ^3^ Division of Endocrinology and Diabetology, Department of Medicine II, Medical Center – University of Freiburg, Faculty of Medicine University of Freiburg Freiburg im Breisgau Germany; ^4^ Department of Pediatrics, Kinderklinik München Schwabing Technical University of Munich School of Medicine Munich Germany; ^5^ Division of Endocrinology and Diabetology, Department of Internal Medicine Medical University of Graz Graz Austria; ^6^ Department of General Pediatrics, Neonatology and Pediatric Cardiology, Medical Faculty and University Hospital Düsseldorf Heinrich Heine University Düsseldorf Düsseldorf Germany; ^7^ Paediatric Endocrinology and Diabetology University Children's Hospital, University of Bern Bern Switzerland; ^8^ AUF DER BULT Diabetes‐Center for Children and Adolescents Hannover Germany; ^9^ Charité – Universitätsmedizin Berlin, corporate member of Freie Universität Berlin and Humboldt‐Universität zu Berlin Sozialpädiatrisches Zentrum, Paediatric Diabetology Berlin Germany; ^10^ Division of Pediatric Endocrinology und Diabetes, Department of Pediatrics University of Heidelberg Heidelberg Germany

**Keywords:** diabetes prospective follow‐up (DPV) registry, MODY, monogenic diabetes, oral antidiabetic drugs, real‐world data

## Abstract

**Background:**

Individuals with maturity‐onset diabetes of the young (MODY) are often misdiagnosed as type 1 or type 2 diabetes and receive inappropriate care. We aimed to investigate the characteristics and treatment of all MODY types in a multicenter, real‐world setting.

**Methods:**

Individuals with MODY from the diabetes prospective follow‐up (DPV) registry were studied. We compared clinical parameters during the first year of diabetes and the most recent treatment year after MODY diagnosis.

**Results:**

A total of 1640 individuals were identified with *GCK*‐MODY (*n* = 941) and *HNF1A*‐MODY (*n* = 417) as the most frequent types. Among these, 912 individuals were available with information during the first and the most recent treatment year (median duration of follow‐up: 4.2 years [2.6–6.6]). Positive beta cell autoantibodies were present in 20.6% (15.2% IAA). Median age at diagnosis ranged from 9.9 years in *GCK*‐MODY (Q1–Q3: 6.2–13.1 years) and *INS*‐MODY (2.7–13.7 years) to 14.3 years (5.0–17.1) in *KCNJ11*‐MODY. Frequency of oral antidiabetic agents (OAD) use increased and insulin decreased in *HNF4A*‐MODY (OAD: 18% to 39%, insulin: 34% to 23%) and in *HNF1A*‐MODY (OAD: 18% to 31%, insulin: 35% to 25%). *ABCC8*‐MODY was characterized by a decrement in nonpharmacological treatment (26% to 16%) and “insulin only” treatment (53% to 42%), while the proportion of individuals treated with OAD but no insulin increased from 0% to 21%.

**Conclusions:**

Our results indicate that some teams caring for individuals with MODY are hesitant with regard to current recommendations. Registries are an essential source of information and provide a basis for discussing treatment guidelines for MODY.

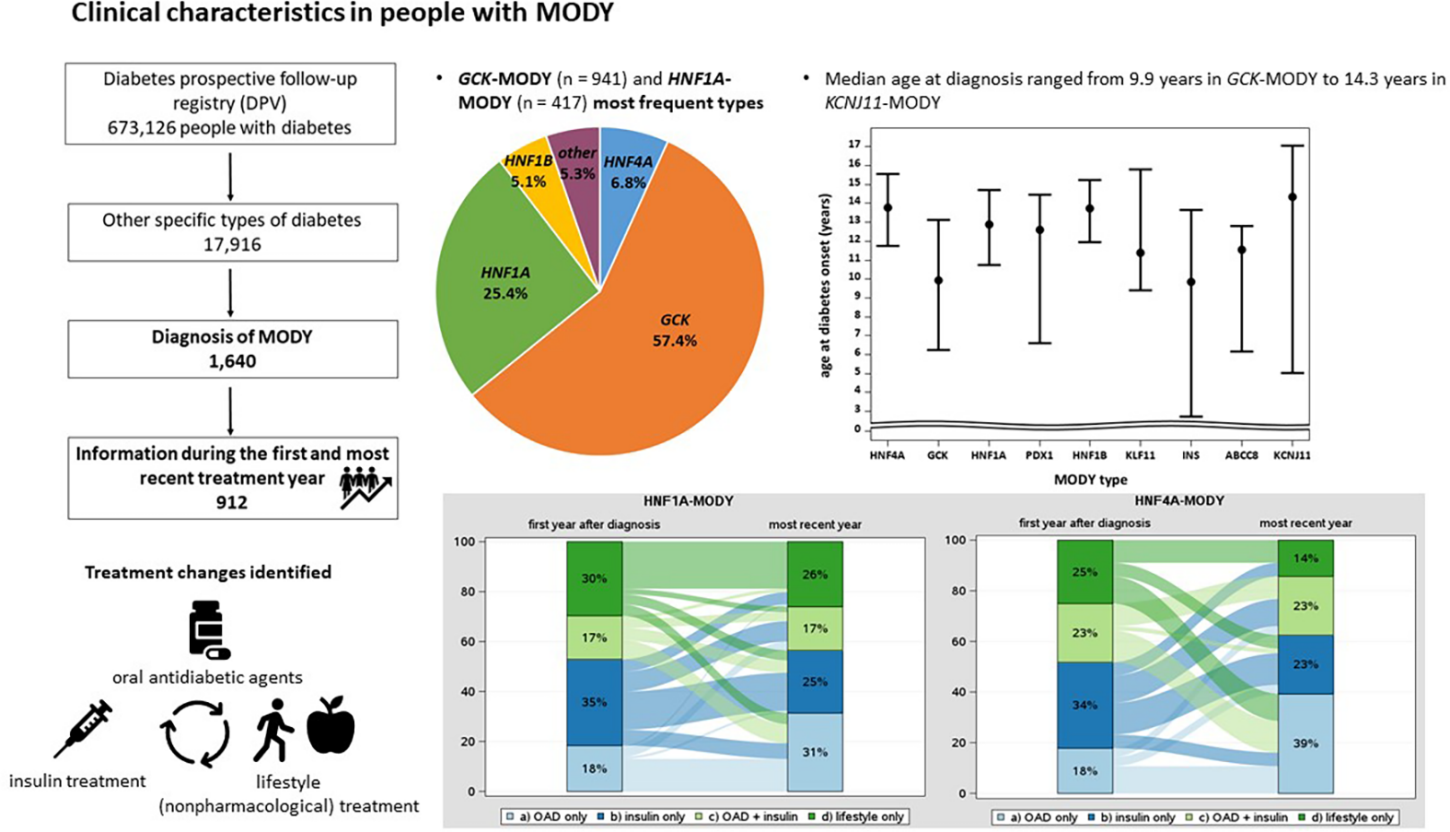

## Graphical abstract

**Highlights**

- A total of 1640 individuals representing all of the 14 identified MODY subtypes were included.
- MODY accounted for 0.2% of children, adolescents, and adults with diabetes in DPV.
- As of today, not all physicians and individuals with MODY are familiar with current treatment recommendations so far.
- Registries are an important source for representing rare diabetes types and provide a basis for discussing treatment guidelines for individuals with MODY.

## 1 INTRODUCTION

The term “maturity‐onset diabetes of the young (MODY)” currently includes 14 known monogenic but clinically heterogeneous forms of diabetes with an autosomal dominant pattern of inheritance.^1^, ^2^ MODY is classified among “other specific types of diabetes” and is characterized by diverse defects of beta‐cell function, representing a small proportion of <5% of individuals with diabetes.^3^, ^4^ Moreover, MODY is represented by a family history of diabetes, diabetes onset before the age of 25 years, mostly impaired insulin secretion with minimal or no defects in insulin action, and absence of islet autoantibodies.^5^ However, de novo mutations in respective genes and positivity of islet autoantibodies have also been reported in association with MODY.^6^, ^7^ With the wide availability of genetic testing, discrimination between functional pathogenic variants and benign or likely benign variants has become more challenging.^8^

Data from diabetes registries have shown that individuals with MODY are often initially misdiagnosed as type 1 or type 2 diabetes.^9^, ^10^, ^11^, ^12^, ^13^, ^14^ Misdiagnosis might lead to inadequate and inefficient treatment.^4^, ^15^ Currently, 14 subtypes of MODY have been identified, which differ in the affected gene, pathogenesis of hyperglycemia, age at onset, recommended treatment, and phenotype.^5^ Pathogenic variants in the glucokinase gene (*GCK*‐MODY), in hepatocyte nuclear factor 1‐alpha (*HNF1A*‐MODY), and *HNF4A* are the most common causes, accounting for >90% of all MODY types in the UK, Europe, and the United States.^16^ *GCK*‐MODY is characterized by mild and nonprogressive hyperglycemia with a low risk of microvascular complications.^17^ Usually, *GCK*‐MODY does not require any pharmacological treatment, as long as there are no other diabetogenic risk factors,^17^ but pregnant women with a *GCK* pathogenic variant might require insulin therapy whether there is increased intrauterine growth/weight gain in the offspring.^18^, ^19^ In contrast, microvascular complications are common in *HNF1A*‐MODY, and current guidelines recommend sulphonylureas as first‐line treatment.^3^, ^20^ Pathogenic variants in *HNF4A* are less common than in *HNF1A*, while the clinical presentation and the response to sulphonylureas are similar to *HNF1A*‐MODY.^4^ However, insulin is the first‐line treatment option for individuals with *HNF1B*‐MODY.^5^, ^16^ A recent study of the diabetes prospective follow‐up (DPV) initiative analyzed individuals with *ABCC8*‐MODY or *KCNJ11*‐MODY and reported a switch from insulin to oral sulfonylureas in most persons while maintaining good metabolic control.^21^

We aimed to investigate the characteristics, frequency of microvascular complications, and cardiovascular risk factors in individuals with MODY in a multicenter, real‐world setting. Moreover, we examined changes in treatment between the first year of diabetes and the most recent treatment year after MODY diagnosis. We studied individuals with all MODY types documented in the DPV registry. In particular, we aimed to compare treatment recommendations for the 14 MODY subtypes with the treatment in real‐world routine clinical care.

## 2 MATERIALS AND METHODS

DPV is a multicenter prospective registry comprising pediatric and adult healthcare facilities.^22^, ^23^, ^24^ As of March 2023, the DPV initiative is represented by 518 centers, including 466 centers in Germany, 46 in Austria, one in Luxembourg, and five in Switzerland. For the current study, 281 centers with documented information on individuals with MODY were included (Supplemental Material). The centers send pseudonymized data to Ulm University every 6 months, where data are validated and subsequently aggregated into the anonymized cumulative DPV registry. Data collection and analysis for benchmarking and diabetes research were approved by the ethics committee of Ulm University (314/21) and by local review boards of the participating centers. The DPV registry was described in more detail elsewhere.^25^

### 2.1 Study population and variables

Individuals of all age groups with a confirmed MODY diagnosis were included in the current analysis. Characteristics extracted from DPV included sex, age, diabetes duration, body mass index (BMI, kg/m^2^), HbA1c (% or mmol/mol), insulin therapy, and daily insulin dose (IU/kg) as well as the use of oral antidiabetic agents (OAD). German reference data for children, adolescents, and adults were used to calculate BMI standard deviation scores (BMI‐SDS).^26^ A BMI‐SDS >1.881 (97th percentile) was defined as obesity. We accounted for different laboratory methods by using the multiple of the mean transformation method to standardize HbA1c values to the Diabetes Control and Complications Trial (DCCT) reference range of 4.05%–6.05% (20.7–42.6 mmol/mol).^27^ Hypertension was specified as a median systolic blood pressure ≥ 140 mmHg and/or a diastolic blood pressure ≥ 90 mmHg or treatment with antihypertensive medication. A total cholesterol ≥200 mg/dL, low‐density lipoprotein cholesterol ≥160 mg/dL, high‐density lipoprotein cholesterol <40 mg/dL, triglyceride ≥150 mg/dL, or treatment with lipid‐lowering medication was specified as dyslipidemia.

We also investigated the proportion of individuals with retinopathy, microalbuminuria (at least two abnormal urine albumin measurements), beta cell antibody positivity, number of positive beta cell antibodies, and pregnancy in females.

DKA was defined as pH less than 7.3 or bicarbonate less than 15 mmol/L^28^ and severe hypoglycemia as an event associated with cognitive impairment requiring help from a third person, including seizure, convulsion, or loss of consciousness.^29^

Whether the documented person or at least one parent was not born in Germany, Austria, Switzerland, or Luxembourg, the individual was considered to have a migratory background.

### 2.2 Aggregation

Aggregated information on clinical parameters during the first year after diabetes diagnosis (excluding the first 3 months for HbA1c), and during the most recent treatment year per person, were compared to show potential switches in treatment and outcome during follow‐up. Treatment was categorized into four groups: OAD/GLP‐1 only, insulin‐only, OAD plus insulin, and lifestyle only (nonpharmacological treatment).

### 2.3 Statistical analysis

Absolute and relative frequencies of documented MODY types were presented overall and stratified by <18 years and ≥18 years of age during the most recent treatment year. In case the absolute frequency of a MODY type was ≥5, clinical characteristics during the first year after diagnosis and the most recent treatment year were compared. Continuous parameters are presented as median together with lower and upper quartiles, and categorical variables are depicted as proportions. DKA and severe hypoglycemia are presented as event rates per 100 person years.

We used Sankey plots to illustrate switches in treatment from the year of diabetes diagnosis to the most recent treatment year. We studied changes in HbA1c and BMI‐SDS from the first year after diagnosis to the most recent treatment year using repeated measures linear regression models. Sex, age at diagnosis, current age, migratory background, and treatment were included as covariables. In addition, an interaction term between year and treatment was included in the models. Regression results are presented as least square means together with 95% confidence intervals (CI).

We used the SAS version 9.4 (TS1M7, SAS Institute Inc., Cary, NC) on a Windows server mainframe for statistical analyses. A two‐sided *p*‐value <0.05 was considered as statistically significant.

## 3 RESULTS

Currently, the DPV registry includes 673 126 individuals with diabetes (164 885 (24.5%) with type 1 diabetes, 464 897 (69%) with type 2 diabetes, and 25 428 (3.8%) with gestational diabetes). In addition, 17 916 (2.7%) individuals are classified as “other specific types of diabetes.” Overall, 1640 individuals harbored a diagnosis of MODY (types 1 to 14) and were included in the analysis.

**FIGURE 1:**
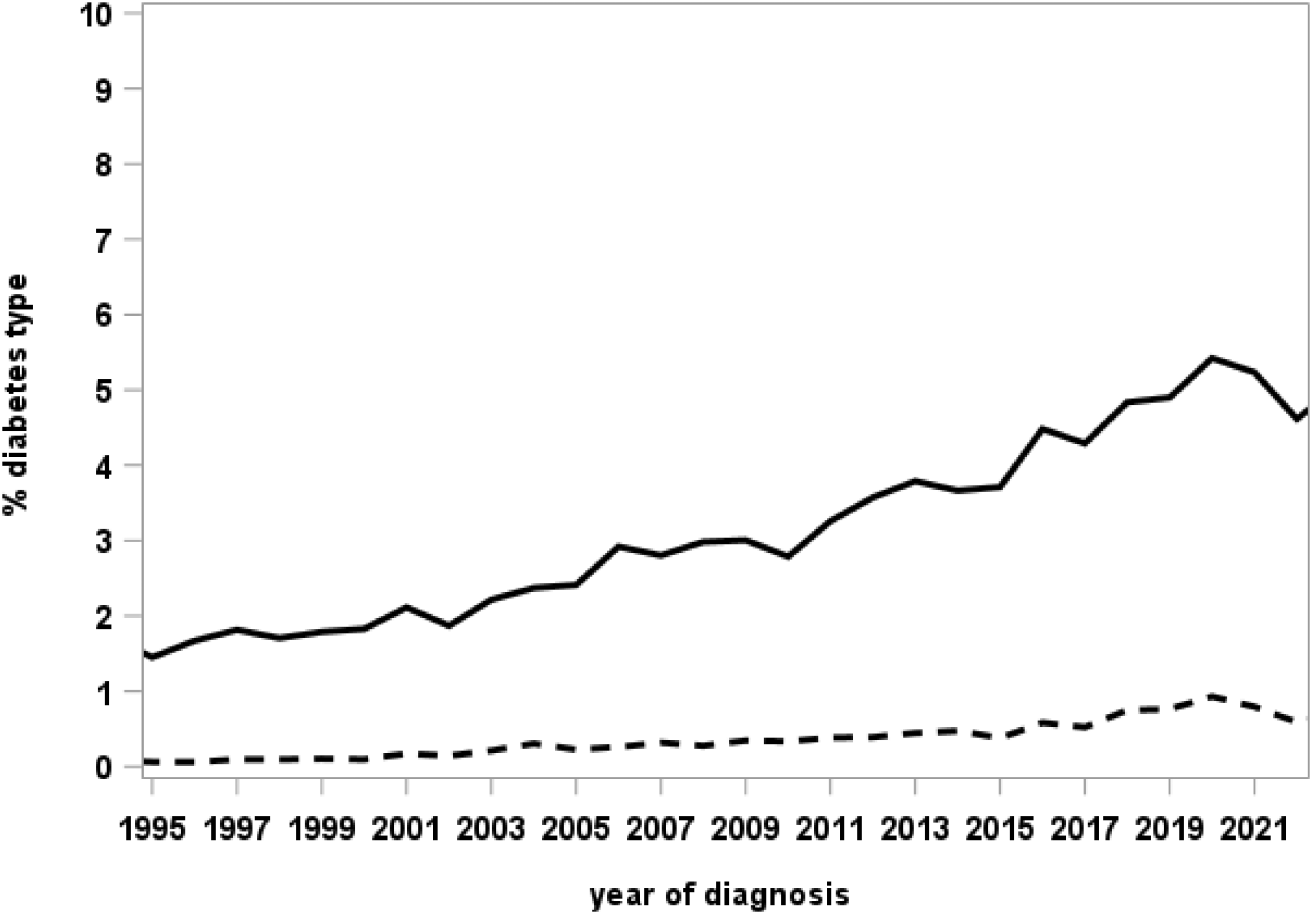
Temporal trend in the proportion of individuals classified with “other specific types of diabetes” (solid line) and MODY (dashed line) by year of diabetes diagnosis. MODY, maturity‐onset diabetes of the young.

Table 1 shows the frequency of the documented MODY types in DPV overall and stratified by age group during the most recent treatment year. All 14 known MODY types are represented in the registry, with *GCK*‐MODY (*n* = 941) and *HNF1A*‐MODY (*n* = 417) as the most frequent types. The proportion of individuals classified as “other specific types of diabetes” increased over time, with a peak of 5.4% of diagnoses in 2020 (of these 0.9% with MODY; Figure 1).

**TABLE 1:** Frequency of MODY types in DPV overall (*n* = 1640) as well as stratified by age group during the most recent treatment year.

MODY type	Gen	Total	<18 years	≥18 years
MODY 1	*HNF4A*	111	58	53
MODY 2	*GCK*	941	795	146
MODY 3	*HNF1A*	417	265	152
MODY 4	*PDX1*	21	13	8
MODY 5	*HNF1B*	84	43	41
MODY 6	*NEUROD1*	3	2	1
MODY 7	*KLF11*	6	4	2
MODY 8	*CEL*	2	2	0
MODY 9	*PAX4*	2	2	0
MODY 10	*INS*	8	4	4
MODY 11	*BLK*	4	3	1
MODY 12	*ABCC8*	30	22	8
MODY 13	*KCNJ11*	10	5	5
MODY 14	*APPL1*	1	1	0

Abbreviation: MODY, maturity‐onset diabetes of the young.

The 912 individuals with MODY with information during the first year after diabetes diagnosis were further investigated, and treatment in the first year after diagnosis and during the most recent treatment year was compared. Minimum time interval was 1 year. The proportion of individuals with a documented MODY type and positive beta cell autoantibodies was 20.6% (15.2% insulin autoantibodies [IAA], 4.2% antibodies to glutamic acid decarboxylase [GAD], 3.0% islet cell antibodies [ICA], and 1.6% IA‐2A). Of those, 15.9% showed one positive antibody only, 3.4% at least two positive antibodies, and 1.3% were the number of positive antibodies was not reported. The frequency of individuals initially diagnosed with another diabetes type was 27.6%; of these, the majority were initially diagnosed with type 1 diabetes (71.8%). The documented individuals with MODY (54.4%) were treated in large diabetes centers, caring for at least 200 persons annually.

Clinical characteristics during the most recent treatment year of the 912 individuals stratified by MODY type are shown in Table 2. Median age at diagnosis ranged from 9.9 years in *GCK*‐MODY (Q1–Q3: 6.2–13.1 years) and *INS*‐MODY (2.7–13.7 years) to 14.3 years (5.0–17.1) in individuals with *KCNJ11*‐MODY. The proportion of children and adolescents <18 years was high, with >50% in all MODY types except for *HNF1B* (44.9%). The proportion of males was highest in *ABCC8*‐MODY (57.9%) and lowest in *HNF4A*‐MODY and *INS*‐MODY (28.6%). During the most recent treatment year, the frequency of insulin therapy was lowest in *GCK*‐MODY (5.7%) and highest in *KLF11*‐MODY (100%), while the proportion of individuals with OAD / GLP‐1 therapy ranged from 6.1% (*GCK*‐MODY) to 62.5% (*HNF4A*‐MODY). Dipeptidyl peptidase‐4 inhibitors (DPP‐4i) were used by 4.1% in individuals with *HNF1B*‐MODY and were less common in the other MODY types. The proportion of sodium‐glucose cotransporter 2 inhibitors (SGLT‐2i) use was 16.7% in *KLF11*‐MODY and 5.3% in *ABCC8*‐MODY, while below 2% in the other types. DKA at diagnosis was absent (0%) in all MODY types except one person with *KLF11*‐MODY (*n* = 1 out of 6, 16.7%). Moreover, event rates of DKA during follow‐up were 0.9 events per 100 person years in *HNF1A*‐MODY and 1.7 events per 100 person years in *HNF1B*‐MODY. Dyslipidemia and hypertension were most common in HNF1B‐MODY (52.9% dyslipidemia, 58.3% hypertension) and KLF11‐MODY (75% dyslipidemia, 100% hypertension), while were less common in *ABCC8*‐MODY (18.2% dyslipidemia, 25.0% hypertension). Proportion of persons with obesity ranged from 0% (*KCNJ11*‐MODY) to 66.7% (*KLF11*‐MODY). The frequency of retinopathy was low, and the presence of microalbuminuria was 0% in *ABCC8‐*MODY and *KCNJ11‐*MODY, while microalbuminuria ranged from 1.8% in *HNF4A*‐MODY and *HNF1A*‐MODY to 16.7% (1 out of 6 individuals) in *KLF11*‐MODY.

**TABLE 2:** Clinical characteristics of the 912 individuals stratified by MODY type (most recent treatment year).

	MODY 1	MODY 2	MODY 3	MODY 4	MODY 5	MODY 7	MODY 10	MODY 12	MODY 13
	HNF4A	GCK	HNF1A	PDX1	HNF1B	KLF11	INS	ABCC8	KCNJ11
	*n* = 56	*n* = 523	*n* = 223	*n* = 13	*n* = 49	*n* = 6	*n* = 7	*n* = 19	*n* = 7
Age at diabetes onset (year)	13.8 (11.8, 15.6)	9.9 (6.3, 13.1)	12.9 (10.7, 14.7)	12.6 (6.6, 14.5)	13.7 (12.0, 15.3)	11.4 (9.4, 15.8)	9.9 (2.7, 13.7)	11.5 (6.2, 12.8)	14.3 (5.0, 17.1)
Age at most recent visit (years)	17.3 (16.3, 20.0)	15.3 (11.7, 17.3)	17.4 (15.7, 18.2)	16.1 (13.7, 18.3)	17.5 (14.9, 19.8)	17.4 (14.1, 18.8)	17.7 (5.5, 19.1)	14.7 (12.0, 18.0)	17.6 (12.4, 20.0)
Diabetes duration (years)	4.0 (2.4, 6.6)	3.8 (2.2, 6.4)	3.8 (2.2, 6.4)	2.9 (2.5, 5.4)	3.4 (2.5, 5.3)	3.4 (2.7, 4.9)	6.5 (2.8, 7.5)	3.8 (2.5, 6.1)	3.3 (2.9, 7.4)
HbA1c during first year (%)	6.2 (5.9, 6.8)	6.2 (5.9, 6.5)	6.0 (5.5, 6.8)	6.0 (5.6, 7.4)	6.0 (5.5, 6.4)	7.6 (7.3, 7.8)	6.9 (6.3, 7.5)	6.1 (5.7, 8.2)	5.7 (5.6, 6.2)
HbA1c at most recent visit (%)	6.7 (6.1, 7.7)	6.2 (5.9, 6.4)	6.5 (5.9, 7.5)	6.5 (5.4, 7.7)	7.2 (5.8, 8.2)	7.2 (6.9, 8.0)	7.2 (6.4, 9.9)	6.5 (6.2, 7.2)	5.7 (5.5, 6.1)
BMI‐SDS	1.2 (0.1, 1.6)	0.3 (−0.5, 1.2)	0.8 (−0.1, 1.6)	1.3 (0.5, 2.4)	0.2 (−0.8, 1.7)	2.2 (1.6, 2.6)	0.3 (−1.0, 1.7)	1.5 (−0.2, 2.3)	−0.4 (−1.0, 0.3)
Daily insulin dose (IU/kg)	0.5 (0.3, 0.8)	0.5 (0.3, 0.9)	0.6 (0.4, 0.9)	0.6 (0.6, 0.9)	0.7 (0.5, 1.1)	0.5 (0.3, 1.1)	0.7 (0.4, 0.8)	0.5 (0.4, 1.0)	0.5 (0.2, 0.7)
Follow‐up (years)	4.5 (2.9, 6.8)	4.1 (2.5, 6.6)	4.2 (2.6, 6.6)	4.0 (2.5, 5.7)	4.0 (2.7, 5.9)	3.8 (3.1, 5.3)	6.4 (2.8, 8.0)	4.3 (2.7, 6.7)	3.7 (3.1, 7.8)
Treatment year	2018 (2013, 2022)	2018 (2013, 2022)	2016 (2011, 2021)	2019 (2019, 2022)	2021 (2017, 2022)	2020 (2018, 2022)	2022 (2020, 2022)	2022 (2019, 2022)	2022 (2015, 2023)
Age < 18 years (%)	64.3	86.4	65.5	69.2	51.0	66.7	42.9	68.4	42.9
Males (%)	28.6	55.4	36.8	38.5	44.9	50.0	28.6	57.9	42.9
Migratory background (%)	17.9	18.5	21.5	61.5	34.7	50.0	42.9	21.1	57.1
DKA at manifestation (%)	0.0	0.0	0.0	0.0	0.0	16.7	0.0	0.0	0.0
DKA at follow‐up (events/100 person years)	0.0	0.0	0.9	0.0	1.7	0.0	0.0	0.0	0.0
Severe hypoglycemia (events/100 person years)	1.7	4.0	3.2	0.0	2.3	0.0	12.9	0.0	0.0
Dyslipidemia (%)	42.1	21.7	43.9	40.0	52.9	75.0	40.0	18.2	0.0
Hypertension (%)	49.1	33.1	52.4	54.5	58.3	100.0	0.0	25.0	71.4
Obesity (%)	16.1	6.7	16.6	30.8	20.4	66.7	14.3	26.3	0.0
Retinopathy (%)	1.8	0.0	0.0	0.0	0.0	0.0	0.0	0.0	0.0
Microalbuminuria (%)	1.8	2.1	1.8	7.7	8.2	16.7	14.3	0.0	0.0
Beta cell antibodies positive (%)	23.2	17.0	22.9	38.5	22.4	50.0	42.9	42.1	28.6
Pregnancy (%)	5.4	1.9	2.7	15.4	0.0	0.0	0.0	5.3	0.0
Insulin therapy (%)	46.4	5.7	42.6	30.8	83.7	100.0	71.4	63.2	28.6
OAD/GLP‐1 therapy (%)	62.5	6.1	48.9	38.5	16.3	33.3	28.6	42.1	42.9
Acarbose (%)	0.0	0.0	0.4	0.0	0.0	0.0	0.0	0.0	0.0
Metformin (%)	25.0	3.8	7.6	15.4	8.2	33.3	0.0	0.0	0.0
DPP‐4i (%)	1.8	0.4	0.4	0.0	4.1	0.0	0.0	0.0	0.0
Glinides (%)	17.9	0.6	21.5	15.4	2.0	0.0	0.0	5.3	28.6
GLP‐1 RA (%)	0.0	0.2	0.9	0.0	2.0	0.0	14.3	5.3	0.0
Glitazones (%)	0.0	0.0	0.0	0.0	0.0	0.0	0.0	0.0	0.0
SGLT‐2i (%)	1.8	0.0	0.4	0.0	0.0	16.7	0.0	5.3	0.0
Sulfonylureas (%)	35.7	2.5	29.1	15.4	2.0	0.0	14.3	36.8	14.3
Lifestyle (%)	14.3	88.9	26.0	38.5	14.3	0.0	28.6	15.8	42.9
Median (Q1–Q3) or %									

Abbreviations: BMI‐SDS, body mass index standard deviation scores; MODY, maturity‐onset diabetes of the young.

### 3.1 Changes in treatment during follow‐up

**FIGURE 2:**
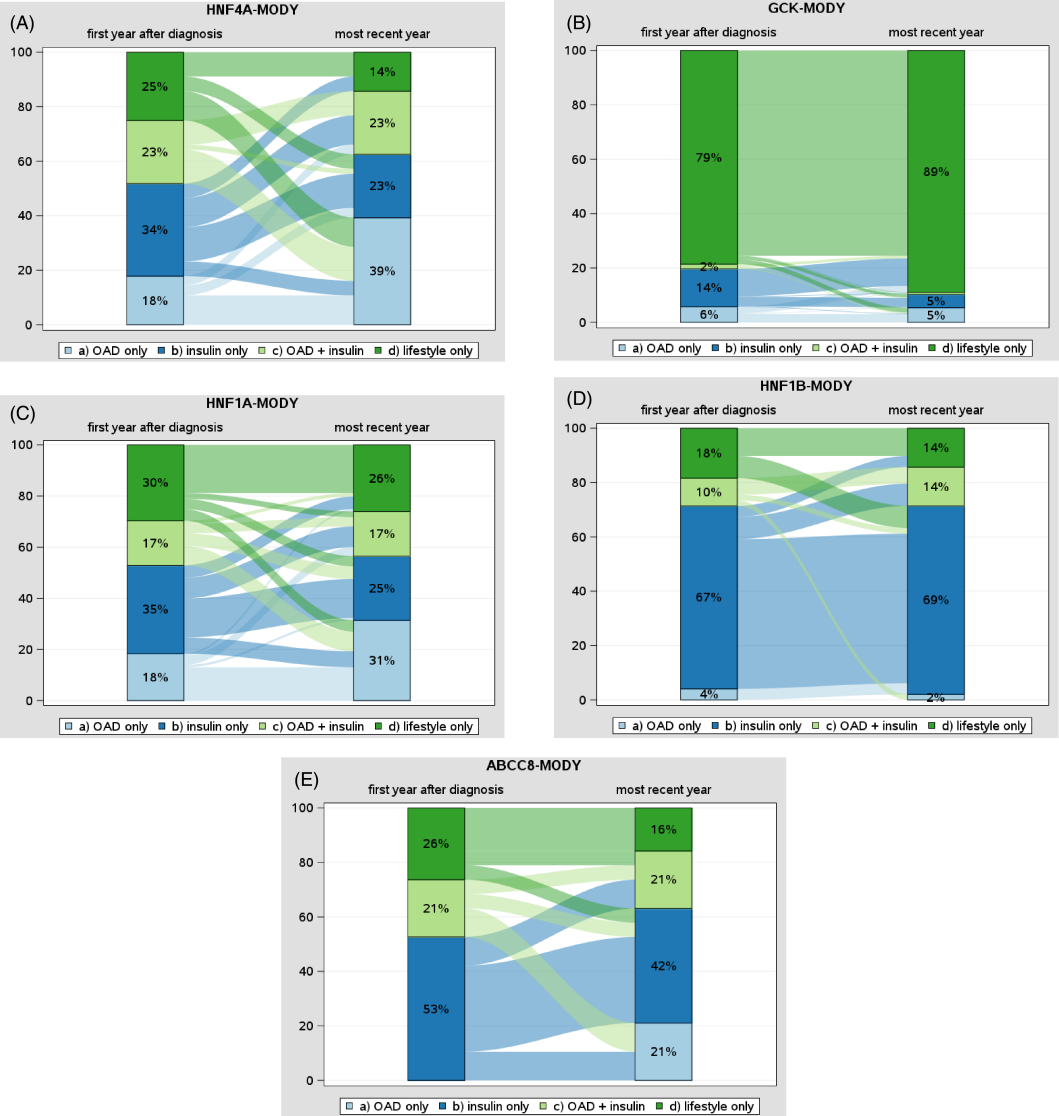
Sankey plots showing changes in treatment from first year after diagnosis to the most recent year in (A) *HNF4A*‐MODY, (B) *GCK*‐MODY, (C) *HNF1A*‐MODY, (D) *HNF1B*‐MODY, and (E) *ABCC8*‐MODY. MODY, maturity‐onset diabetes of the young.

Changes in treatment for the MODY types *HNF4A*, *GCK*, *HNF1A*, *HNF1B*, and *ABCC8* from the first year after diagnosis to the most recent treatment year are presented in Figure 2A–E. Median time from the first year to the most recent treatment year was 4.2 years (2.6–6.6).

In individuals with *HNF4A*‐MODY, we observed a shift from lifestyle‐only and insulin‐only towards treatment with OAD from the first year after diagnosis to the most recent year (Figure 2A). For example, the use of OAD only increased from 18% to 39%, while lifestyle‐only decreased from 25% to 14% and insulin‐only from 34% to 23%.

With regard to *GCK*‐MODY, the majority of individuals were on lifestyle therapy only (89% in the most recent treatment year; Figure 2B). The proportion of individuals with *HNF1A*‐MODY treated with OAD only increased over time from 18% to 31%, while treatment with insulin‐only was 35% in the first year after diagnosis and 25% in the most recent treatment year (Figure 2C). Individuals with *HNF1B*‐MODY showed a high proportion of treatment with insulin alone in both periods, with 67% in the first year and 69% in the most recent treatment year (Figure 2D). *ABCC8*‐MODY was characterized by a decrement in lifestyle‐only (26% to 16%) and insulin‐only (53% to 42%), while the proportion of individuals treated with OAD only increased to 21% (Figure 2E). Changes in treatment for *PDX1*‐MODY, *KLF11*‐MODY, and *KCNJ11*‐MODY are presented in Supplemental Figure 1.

### 3.2 Glycemic control and BMI‐SDS

Changes in adjusted HbA1c means were studied for the MODY types *HNF4A*, *GCK*, *HNF1A*, *HNF1B*, *ABCC8*, and *KCNJ11*, while *ABCC8* and *KCNJ11* were combined. Overall, HbA1c differed between the treatment groups (Table 3). However, we observed no statistically significant changes in HbA1c from the first to the most recent treatment year in *HNF4A*‐MODY, *HNF1B*‐MODY, and *ABCC8*‐MODY/*KCNJ11*‐MODY within the treatment groups. In individuals with *GCK*‐MODY, HbA1c increased slightly from 6.4% (95% CI: 6.3–6.5) to 6.8% (6.6–6.9) in the group treated with insulin‐only. Moreover, we found a significant increase in HbA1c in *HNF1A* in the insulin‐only (6.5% [6.2–6.8] to 7.5% [7.1–7.8]) and in the OAD and insulin (6.6% [6.2–7.0] to 7.6% [7.1–8.0]) group. No significant changes from the first to the most recent treatment year in BMI‐SDS were observed.

**TABLE 3:** Changes in HbA1c and BMI‐SDS from first year to most recent treatment year, adjusted for sex, age at diagnosis, current age, migratory background, and treatment.

Treatment	HbA1c			BMI‐SDS		
	Adjusted mean (95%‐confidence interval)		*p*‐value	Adjusted mean (95%‐confidence interval)		*p*‐value
	First year	Most recent year		First year	Most recent year	
*HNF4A*‐MODY						
OAD only	6.3 (5.3–7.3)	7.0 (6.4–7.6)	0.242	1.0 (0.4–1.5)	0.9 (0.5–1.3)	0.876
Insulin only	7.0 (6.3–7.7)	7.6 (6.8–8.5)	0.254	0.9 (0.4–1.3)	1.1 (0.6–1.7)	0.388
OAD plus insulin	6.6 (5.8–7.4)	6.8 (6.1–7.6)	0.682	1.0 (0.5–1.4)	1.0 (0.5–1.5)	0.909
Lifestyle only	6.4 (5.5–7.3)	6.5 (5.4–7.6)	0.901	0.6 (0.1–1.2)	1.1 (0.4–1.7)	0.249
*GCK*‐MODY						
OAD only	6.3 (6.1–6.5)	6.2 (6.1–6.4)	0.499	0.6 (0.3–0.9)	0.6 (0.3–0.9)	0.937
Insulin only	6.4 (6.3–6.5)	6.8 (6.6–6.9)	<0.001	0.0 (−0.3–0.2)	0.1 (−0.2–0.5)	0.343
OAD plus insulin	5.9 (5.6–6.3)	6.0 (5.6–6.4)	0.864	0.8 (0.3–1.4)	0.8 (0.0–1.5)	0.921
Lifestyle only	6.2 (6.1–6.2)	6.1 (6.1–6.2)	0.282	0.1 (0.0–0.3)	0.2 (0.1–0.3)	0.230
*HNF1A*‐MODY						
OAD only	6.0 (5.6–6.4)	6.6 (6.2–6.9)	0.026	0.8 (0.5–1.0)	0.8 (0.6–1.0)	0.882
Insulin only	6.5 (6.2–6.8)	7.5 (7.1–7.8)	<0.001	0.7 (0.5–0.9)	0.8 (0.6–1.1)	0.346
OAD plus insulin	6.6 (6.2–7.0)	7.6 (7.1–8.0)	0.001	0.8 (0.5–1.0)	0.9 (0.6–1.1)	0.623
Lifestyle only	5.9 (5.5–6.3)	6.2 (5.9–6.6)	0.143	0.5 (0.3–0.8)	0.6 (0.3–0.8)	0.863
*HNF1B*‐MODY						
OAD only	6.3 (3.7–8.8)	7.2 (3.6–10.7)	0.679	0.0 (−1.1–1.2)	−0.7 (−2.4–0.9)	0.447
Insulin only	6.5 (5.7–7.3)	7.2 (6.6–7.9)	0.179	0.5 (0.1–1.0)	0.3 (−0.1–0.7)	0.334
OAD plus insulin	7.6 (6.0–9.3)	8.0 (6.6–9.5)	0.718	0.6 (−0.2–1.4)	0.9 (0.1–1.6)	0.579
Lifestyle only	6.3 (4.7–7.9)	5.9 (4.4–7.3)	0.646	0.5 (−0.3–1.4)	0.4 (−0.4–1.2)	0.760
*ABCC8*‐/*KCNJ11*‐MODY						
OAD only	NA	6.5 (5.8–7.2)	NA	NA	0.3 (−0.5–1.0)	NA
Insulin only	6.6 (5.9–7.2)	6.8 (6.1–7.5)	0.579	1.3 (0.5–2.1)	0.5 (−0.4–1.5)	0.172
OAD plus insulin	7.0 (6.1–7.8)	7.4 (6.6–8.1)	0.341	0.7 (0.0–1.5)	1.1 (0.4–1.9)	0.282
Lifestyle only	6.2 (5.5–7.0)	6.1 (5.3–6.9)	0.620	1.1 (0.2–2.0)	0.6 (−0.2–1.5)	0.081

Abbreviations: BMI‐SDS, body mass index standard deviation scores; MODY, maturity‐onset diabetes of the young; NA, not applicable; OAD, oral antidiabetic agents.

## 4 DISCUSSION

We identified 1640 individuals with MODY in the DPV registry, with *GCK* (*n* = 941) and *HNF1A* (*n* = 417) as the most common MODY types. Of these, 912 individuals presented information during the first year after diagnosis of diabetes and the most recent treatment year. Molecular‐genetic classified MODY accounted for 0.2% of children, adolescents, and adults with diabetes in DPV, and therefore, our presented prevalence is lower in comparison to the reported prevalences for children (0.9%) and adults (0.4%) in population‐based studies from Norway.^13^, ^30^ However, a previous study using data from the DPV registry identified 0.8% of children and adolescents below 20 years of age with MODY.^9^ In accordance with our research, Kropff and colleagues observed a MODY prevalence of 0.2% in young adults in their community‐based, cross‐sectional study in the UK.^31^ Studies with a systematic genetic screening approach found prevalences of 2.5% to 4.2% of all diabetes cases in children and adolescents in Western European cohorts.^32^, ^33^

Most MODY cases currently documented in DPV were diagnosed between 2007 and 2020, indicating an increased awareness of MODY during the last 15 years and an improvement in clinical assessment and genetic analysis.^21^, ^34^ Further, the growing recognition of the clinical significance of MODY might improve screening and diagnostic procedures.^34^

Positive beta cell autoantibodies were found in 20.6% (15.2% insulin autoantibodies [IAA], 4.2% antibodies to glutamic acid decarboxylase [GAD], 3.0% islet cell antibodies [ICA], and 1.6% IA–2A). Of those, 15.9% showed one positive antibody, 3.4% had at least two positive antibodies, and 1.3% were reported with an unknown number of positive antibodies. A previous study of the DPV initiative showed a slightly lower proportion of positive beta‐cell autoantibodies in individuals with MODY (17%).^9^ However, we included all age groups in our analysis, whereas Schober and colleagues studied children and adolescents <20 years only. Further, 25% of individuals with MODY were positive for GAD and IA‐2 in a small study from the Czech Republic.^7^ It has to be noted that these might be transient beta cell antibodies that are induced by beta cell distress and are therefore reversible.

No DKA at diagnosis was observed in a study conducted in Finland.^35^ In our analysis, only one patient with *KLF11*‐MODY had DKA at diagnosis. The frequency of microvascular complications was reported to be rare in *GCK*‐MODY.^17^, ^36^ Steele and colleagues observed microvascular complications in 1% of individuals with *GCK* in a cross‐sectional study in the UK.^17^ The proportion of microalbuminuria (at least two abnormal urine albumin measurements) was around 2% in *HNF4A*, *GCK*, and *HNF1A*, whereas one patient representing 16.7% with *KLF11‐MODY* presented with microalbuminuria in our analyses.

In accordance with a study from Poland,^37^ retinopathy was not detected in *GCK*‐MODY in our analysis but in 1.8% of individuals with *HNF4A*‐MODY. One of the main findings of a single‐center study in India was that the prevalence of retinopathy and nephropathy in MODY was higher compared to type 1 and type 2 diabetes.^38^ Therefore, regular screening for retinopathy and nephropathy in individuals with MODY, especially with variants in *HNF4A*, *HNF1A*, and *HNF1B*, is recommended.

For the MODY types *HNF4A* and *HNF1A*, we observed a shift towards treatment with OAD during the most recent treatment year. Sulfonylureas are recommended as first‐line therapy,^3^, ^20^ and accordingly, 36% of documented individuals in DPV with *HNF4A* and 29% with *HNF1A* were treated with sulfonylureas in the most recent treatment year. In the case of *HNF1A*, the risk of hypoglycemia must be considered, as insulin sensitivity can be normal or increased in individuals with *HNF1A*‐MODY.^36^ Glinides and glucagon‐like Peptide‐1 receptor agonists have also been effective in *HNF4A‐*MODY and *HNF1A‐*MODY.^39^, ^40^ Current guidelines do not recommend pharmacological treatment for *GCK*‐MODY, except during pregnancy.^3^, ^20^ The proportion of pregnancy in females in the most recent treatment year was 1.9% in our study, and 89% of all persons with GCK did not take any pharmacological treatment. Insulin treatment played a significant role in *HNF1B*‐MODY, as 69% were on insulin at the most recent treatment year. The *HNF1B* mutation is associated with a heterogeneous phenotype, also known as “renal cysts and diabetes” syndrome.^4^, ^5^ Early initiation of insulin treatment is recommended due to decreased insulin secretion with progressive worsening of glucose control.^5^ Treatment with OAD only increased to 21% in individuals with *ABCC8*‐MODY, while the proportion with insulin‐only (42%) and OAD plus insulin (21%) was high. Variants in the potassium channel (*ABCC8 or KCNJ11*) are often associated with neonatal diabetes, but the onset of diabetes can also occur later in life.^5^ Variants in the *ABCC8* gene were more common in DPV compared to *KCNJ11*.^21^ Studies have shown that *ABCC8* is often misdiagnosed and unnecessarily treated with insulin, while sulfonylureas are primarily recommended.^5^ Our study's high proportion of insulin treatment might indicate that not all physicians are familiar with these current recommendations. Patients may also be hesitant to stop insulin replacement. Similarly, sulfonylureas are also recommended for *KCNJ11‐*MODY.

*PDX1*‐MODY is rare and was observed in 13 persons with information during the first and most recent treatment year in DPV. *PDX1* is characterized by a mild form of diabetes and can usually be treated with OAD.^5^ Accordingly, we found a proportion of 39% with OAD and 31% with insulin therapy. Furthermore, we identified six persons with a *KLF11* variant with follow‐up information (100% with insulin) and seven with variants in the *INS* gene (29% with OAD, 71% with insulin).

Changes in HbA1c from the first year after diagnosis to the most recent treatment year were studied for the MODY types *HNF4A*, *GCK*, *HNF1A*, *HNF1B*, and *ABCC8*/*KCNJ11*. HbA1c differed between the treatment groups, showing higher HbA1c values with insulin treatment. We can assume that insulin is initiated in case of poor glycemic control. Increases in HbA1c from the first to the most recent treatment year were only observed in *GCK*‐MODY and *HNF1A*‐MODY, while the other MODY types showed no significant changes in glycemic control during follow‐up. In *HNF1A*, HbA1c increased from 6.5% (6.2–6.8) to 7.5% (7.1–7.8), in line with a literature review showing that glycemic control worsens over time in *HNF1A*‐MODY.^34^

Clinical presentation, treatment, and outcomes among families with MODY can be quite heterogeneous despite the same underlying mutation.^5^, ^34^ For example, the heterogeneity in MODY across families can be influenced by genetic factors, environmental factors, healthcare management, and psychosocial factors.

Due to low numbers, only descriptive analyses for some MODY types were conducted, and no further statistical analyses were possible, limiting our study's scope. Moreover, we assume an underreporting of MODY in DPV as studies using a systematic genetic screening approach found higher prevalences of MODY.^32^, ^33^ Detailed documentation, including genetic data, is important for treatment and outcome of individuals with MODY. However, 14 subtypes of MODY are currently identified, and all subtypes are represented in the DPV registry. Therefore, this dataset is one of the most comprehensive, including common and rare MODY forms. A further strength of our analysis is that the data are generated from a standardized data collection network, representing a multicenter, real‐world setting. DPV is representative of pediatric diabetes care and adults with diabetes treated in diabetes‐specialized practices in Germany.

MODY identification is essential regarding personalized treatment and screening of family members. We observed a proportion of 27.6% initially diagnosed as another diabetes type, potentially leading to inadequate treatment. Accordingly, current guidelines recommend genetic testing for MODY in children and young adults without typical characteristics of type 1 and type 2 diabetes (e.g., negative diabetes–associated autoantibodies or no obesity) and a family history of diabetes, suggesting an autosomal dominant inheritance pattern.^3^, ^20^ Because a MODY diagnosis has important implications for treatment and outcome, Johansson and colleagues suggest including molecular screening for the most common MODY genes for all antibody‐negative children in routine diagnostics. However, we and others have shown that MODY can also be present in persons with positive beta cell autoantibodies.^7^ Nevertheless, according to national and international guidelines, the detection of autoimmunity leads to the diagnosis of type 1 diabetes.^3^, ^20^ It remains unclear whether a single positive antibody titer is sufficient or whether—similar to stage 1 or 2 of type 1 diabetes, at least two titers are required. Antibodies associated with diabetes as a surrogate parameter for beta cell destruction seem to exist for reasons beyond autoimmunity and are found in 10%–20% for persons with clinical type 2 diabetes.^41^, ^42^, ^43^

## 5 CONCLUSIONS

Results of the DPV registry indicate an increased awareness of MODY during the last 15 years and an improvement in clinical assessment and genetic analysis. However, we assume an underreporting of MODY in DPV as studies using a systematic genetic screening approach found higher prevalences of MODY. As of today, some physicians and individuals with MODY are hesitant to change treatment according to current treatment recommendations Registries are essential for representing rare diabetes types and provide a basis for discussing treatment guidelines for individuals with MODY.

## FUNDING INFORMATION

Financial support for DPV was provided by the German Center for Diabetes Research (DZD, grant number 82DZD14E03) and by the Robert Koch Institute (RKI, grant number 1368‐1711). Additional funding was provided by the REDDIE project (grant agreement 101095556). Sponsors were not involved in data acquisition or analysis.

## CONFLICT OF INTEREST STATEMENT

The authors declare no conflicts of interest.

## Supporting information


**Data S1.** Supporting Information.
