# Supplementary material for: Clinical characteristics, treatment, and treatment switch after molecular‐genetic classification in individuals with maturity‐onset diabetes of the young: Insights from the multicenter real‐world DPV registry
Source: J Diabetes. 2024 Nov 7;16(11):e70028. doi: 10.1111/1753-0407.70028 (PMC11544032; doi:10.1111/1753-0407.70028)
Supplement: Supplementary file 1 — Data S1. Supporting Information. [file JDB-16-e70028-s001.docx]

**Supplemental Material**

**Clinical characteristics, treatment and treatment switch after molecular-genetic classification in people with Maturity-onset Diabetes of the Young: Insights from the multicenter real-world DPV registry**

Stefanie Lanzinger^1,2^, Katharina Laubner^3^, Katharina Warncke^4^, Julia K. Mader^5^, Sebastian Kummer^6^, Claudia Boettcher^7^, Torben Biester^8^, Angela Galler^9^, Daniela Klose^10^, Reinhard W. Holl^1,2^

^1^Institute of Epidemiology and Medical Biometry, CAQM, Ulm University, Ulm, Germany
^2^German Center for Diabetes Research (DZD), Munich-Neuherberg, Germany **^3^**Division of Endocrinology and Diabetology, Department of Medicine II, Medical Center - University of Freiburg, Faculty of Medicine, University of Freiburg, Freiburg im Breisgau, Germany
^4^Department of Pediatrics, Kinderklinik München Schwabing, Technical University of Munich School of Medicine, Munich, Germany
^5^Division of Endocrinology and Diabetology, Department of Internal Medicine, Medical University of Graz, Graz, Austria
^6^Department of General Pediatrics, Neonatology and Pediatric Cardiology, Medical Faculty and University Hospital Düsseldorf, Heinrich Heine University Düsseldorf, Germany
**^7^**Paediatric Endocrinology and Diabetology, University Children's Hospital, University of Bern, Bern, Switzerland
^8^AUF DER BULT, Diabetes-Center for Children and Adolescents, Hannover, Germany
^9^Charité - Universitätsmedizin Berlin, corporate member of Freie Universität Berlin and Humboldt-Universität zu Berlin, Sozialpädiatrisches Zentrum, Paediatric Diabetology, Berlin, Germany
^10^University of Heidelberg, Department of Pediatrics, Division of Pediatric Endocrinology und Diabetes, Heidelberg, Germany

List of DPV centers contributing data to the analyses:

Aachen - Innere RWTH, Aachen - Uni-Kinderklinik RWTH, Aalen Kinderklinik, Ahlen St. Franziskus Kinderklinik, Aidlingen Praxisgemeinschaft, Amstetten Klinikum Mostviertel Kinderklinik, Arnsberg-Hüsten Karolinenhosp. Kinderabteilung, Aue Helios Kinderklink, Augsburg IV. Med. Uni-Klinik, Augsburg Josefinum Kinderklinik, Augsburg Uni-Kinderklinik, Aurich Kinderklinik, Bad Aibling Internist. Praxis, Bad Kreuznach Diakonie Kikli, Bad Kösen Median Kinderklinik, Bad Mergentheim - Diabetesfachklinik, Bad Mergentheim - Kinderdiabetologische Praxis, Bad Oeynhausen Herz-und Diabeteszentrum NRW, Bad Orb Spessart Klinik, Bad Waldsee Kinderarztpraxis, Bautzen Oberlausitz KK, Bayreuth Innere Medizin, Berlin DRK-Kliniken Pädiatrie, Berlin Endokrinologikum, Berlin Klinik St. Hedwig Innere, Berlin Lichtenberg - Kinderklinik, Berlin Schlosspark-Klinik Innere, Berlin Virchow-Kinderklinik, Berlin Vivantes Hellersdorf Innere, Bern Inselspital Kinderklinik, Bielefeld Kinderklinik Gilead, Bielefeld Pädiatrisches Forum, Bocholt Kinderklinik, Bochum Universitätskinderklinik St. Josef, Bodnegg - MVZ Wollmarshöhe, Bonn Uni-Kinderklinik, Braunschweig Kinderarztpraxis, Bremen - Kinderklinik Nord, Bremen - Mitte Innere, Bremen Zentralkrankenhaus Kinderklinik, Bremerhaven Kinderklinik, Bruchweiler Edelsteinklinik Kinder-Reha, Böblingen Kinderklinik, Celle Klinik für Kinder- und Jugendmedizin, Chemnitz Kinderklinik, Coburg Innere Medizin, Coesfeld Kinderklinik, Darmstadt Kinderklinik Prinz. Margaret, Datteln Vestische Kinderklinik, Deggendorf Medizinische Klinik II, Deggendorf Pädiatrie-Praxis, Dornbirn Kinderklinik, Dortmund Kinderklinik, Dortmund Knappschaftskrankenhaus Innere, Dortmund-St. Josefshospital Innere, Dresden Neustadt Kinderklinik, Dresden Uni-Kinderklinik, Duisburg Homberg Helios Rhein-Ruhr Kliniken GmbH, Duisburg Sana Kinderklinik, Duisburg St. Anna Innere Helios Rhein-Ruhr Kliniken GmbH, Duisburg-St.Johannes Helios, Düren-Birkesdorf Kinderklinik, Düsseldorf Uni-Kinderklinik, Eckernförde Gem.-Prax, Erfurt Kinderklinik, Erlangen Uni Innere Medizin, Erlangen Uni-Kinderklinik, Essen Diabetes-Schwerpunktpraxis, Essen Elisabeth Kinderklinik, Essen Uni-Kinderklinik, Esslingen Klinik für Kinder und Jugendliche, Eutin Kinderklinik, Feldkirch Kinderklinik, Forchheim Diabeteszentrum SPP, Frankfurt Diabeteszentrum Rhein-Main-Erwachsenendiabetologie (Bürgerhospital), Frankfurt Diabeteszentrum Rhein-Main-pädiat. Diabetologie (Clementine-Hospital), Frankfurt Uni-Kinderklinik, Frankfurt Uni-Klinik Innere2, Frankfurt-Sachsenhausen Innere, Frankfurt-Sachsenhausen Innere MVZ, Freiburg Uni Innere, Freiburg Uni-Kinderklinik, Friedberg Innere Klinik, Gaissach Fachklinik der Deutschen Rentenversicherung Bayern Süd, Garmisch-Partenkirchen Kinderklinik, Gelnhausen Kinderklinik, Gelsenkirchen Kinderklinik Marienhospital, Gera Kinderklinik, Gießen Uni-Kinderklinik, Graz Uni-Kinderklinik, Greifswald Uni-Kinderklinik, Gummersbach Oberbergklinikum, Göppingen Kinderklinik am Eichert, Göttingen Uni Gastroenterologie, Göttingen Uni-Kinderklinik, Hagen Kinderklinik, Halle Uni-Kinderklinik, Halle-Dölau Städtische Kinderklinik, Hamburg Altonaer Kinderklinik, Hamburg Endokrinologikum, Hamburg Kinderklinik Wilhelmstift, Hamburg-Nord Kinder-MVZ, Hameln Kinderklinik, Hamm Kinderklinik, Hanau Kinderklinik, Hanau diabetol. Schwerpunktpraxis, Hannover Kinderklinik auf der Bult, Haren Kinderarztpraxis, Heide Kinderklinik, Heidelberg St. Josefskrankenhaus, Heidelberg Uni-Kinderklinik, Heidenheim Kinderklinik, Heilbronn Innere Klinik, Heilbronn Kinderklinik, Herdecke Kinderklinik, Herford Kinderarztpraxis, Herford Klinikum Kinder & Jugendliche, Hermeskeil Kinderpraxis, Hildesheim Bernward Krks Kinderheilkunde, Hildesheim GmbH - Innere, Hildesheim Kinderarztpraxis, Hinrichsegen-Bruckmühl Diabetikerjugendhaus, Hof Kinderklinik, Homburg Uni-Kinderklinik Saarland, Innsbruck Uni-Kinderklinik, Iserlohn Innere Medizin, Itzehoe Kinderklinik, Jena Kinderarztpraxis, Jena Uni-Kinderklinik, Kaiserslautern Kinderarztpraxis, Kaiserslautern-Westpfalzklinikum Kinderklinik, Kamen Klinikum Westfalen Hellmig Krankenhaus, Karlsburg Klinik für Diabetes & Stoffwechsel, Karlsruhe Schwerpunktpraxis, Karlsruhe Städtische Kinderklinik, Kassel Klinikum Kinder- und Jugendmedizin, Kaufbeuren Kinderklinik, Kiel Städtische Kinderklinik, Kiel Universitäts-Kinderklinik, Kirchen DRK Krankenhaus Kinderklinik, Kirchheim-Nürtingen Innere, Klagenfurt Kinderklinik, Koblenz Kinderklinik Kemperhof, Konstanz Kinderklinik, Krefeld Innere Klinik, Kreischa-Zscheckwitz Klinik Bavaria, Köln Kinderklinik Amsterdamerstrasse, Köln Uni-Kinderklinik, Landau Innere, Landshut Kinderklink, Leer Klinikum - Klinik Kinder & Jugendmedizin, Leipzig Uni-Kinderklinik, Leoben LKH Kinderklinik, Leverkusen Kinderklinik, Limburg Innere Medizin, Lingen Kinderklinik St. Bonifatius, Linz KUK MedCampus IV Kinderklinik, Linz Krankenhaus der Barmherzigen Schwestern Kinderklinik, Ludwigsburg Kinderklinik, Ludwigshafen Kinderklinik St.Anna-Stift, Luxembourg - Centre Hospitalier, Lübeck Uni-Kinderklinik, Lübeck Uni-Klinik Innere Medizin, Lüdenscheid Märkische Kliniken - Kinder & Jugendmedizin, Magdeburg Ki-Klinik St. Marienstift, Magdeburg Städtisches Klinikum Innere, Magdeburg Uni-Kinderklinik, Mainz Uni-Kinderklinik, Mannheim Uni-Kinderklinik, Marburg Uni-Kinderklinik, Mechernich Kinderklinik, Meissen Kinderklinik Elblandklinikum, Memmingen Internistische Praxis, Minden Kinderklinik, Moers Kinderklinik, Murnau am Staffelsee - diabetol. SPP, Mutterstadt Kinderarztpraxis, Mödling Kinderklinik, Mönchengladbach Kinderklinik Rheydt Elisabethkrankenhaus, Mühlacker Enzkreiskliniken Innere, München 3. Orden Kinderklinik, München Kinderarztpraxis diabet. SPP, München Praxiszentrum Saarstrasse, München von Haunersche Kinderklinik, München-Gauting Kinderarztzentrum, München-Schwabing Kinderklinik, Münster Herz Jesu Innere, Münster Ludgerus-Kliniken GmbH, Münster pädiat. Schwerpunktpraxis, Neuburg Kinderklinik, Neumarkt Innere, Neunkirchen Marienhausklinik Kohlhof Kinderklinik, Neuss Lukas-Krankenhaus Kinderklinik, Neuss Lukaskrankenhaus Kinderklinik, Neuwied Kinderklinik Elisabeth, Nürnberg Cnopfsche Kinderklinik, Nürnberg Uniklinik Zentrum f Neugeb./Kinder & Jugendl., Oberhausen Kinderklinik, Oberhausen Kinderpraxis, Oberwart - Burgenländische Krankenanstalten Pädiatrie, Offenburg Kinderklinik, Oldenburg Kinderklinik, Oldenburg Schwerpunktpraxis Pädiatrie, Olpe pädiatrische Gemeinschaftspraxis, Oschersleben MEDIGREIF Bördekrankenhaus, Osnabrück Christliches Kinderhospital, Paderborn St. Vincenz Kinderklinik, Papenburg Marienkrankenhaus Kinderklinik, Pforzheim Kinderklinik, Plauen Vogtlandklinikum, Prien am Chiemsee Innere, Rastatt Kreiskrankenhaus Innere, Ravensburg Kinderklink St. Nikolaus, Regensburg Kinderklinik St. Hedwig, Reutlingen Kinderarztpraxis, Reutlingen Kinderklinik, Reutlingen Klinikum Steinenberg Innere, Rheine Mathiasspital Kinderklinik, Rosenheim Innere Medizin, Rosenheim Kinderklinik, Rosenheim Schwerpunktpraxis, Rostock Uni-Kinderklinik, Rostock Universität Innere Medizin, Rotenburg/Wümme Agaplesion Diakonieklinikum Kinderabteilung, Rüsselsheim Kinderklinik, Rüsselsheim MVZ, Saaldorf-Surheim Diabetespraxis, Saalfeld Thüringenklinik Kinderklinik, Salzburg Universitäts-Kinderklinik, Scheidegg Prinzregent Luitpold, Schleswig Heliosklinik Kinderklinik, Schw. Gmünd Stauferklinik Kinderklinik, Schweinfurt Kinderklinik, Schwerin Kinderklinik, Schwäbisch Hall Diakonie Kinderklinik, Siegen Kinderklinik, Singen Hegau Bodensee-Klinikum Kinderklinik, Singen Kinderarztpraxis, Spaichingen Innere, Speyer Diakonissen Stiftungskrankenhaus Pädiatrie, St. Augustin Kinderklinik, St. Gallen Ostschweizer Kinderspital, St. Pölten Universitäts-Kinderklinik, Stade Kinderklinik, Stolberg Kinderklinik, Stuttgart Olgahospital Kinderklinik, Sylt Rehaklinik, Timmendorfer Strand, Traunstein Kinderklinik, Trier Kinderklinik der Borromäerinnen, Trostberg Innere, Tübingen Uni-Kinderklinik, Ulm Endokrinologikum Amedes, Ulm Uni-Kinderklinik, Vechta Kinderklinik, Viersen Kinderkrankenhaus St. Nikolaus, Villach Kinderklinik, Villingen-Schwenningen Schwarzwald Baar Klinikum Kinderklinik, Volkertshausen Gemeinschaftspraxis, Vöcklabruck Kinderklinik, Waldshut Kinderpraxis, Wangen Oberschwabenklinik Innere Medizin, Waren-Müritz Kinderklinik, Weingarten Kinderarztpraxis, Weisswasser Kreiskrankenhaus, Wesel Marienhospital Kinderklinik, Wien KH Nord-Klinik Floridsdorf, Wien Preyersches Kinderspital, Wien SMZ Ost Donauspital, Wien Uni-Kinderklinik, Wiesbaden Helios Horst-Schmidt-Kinderkliniken, Wiesbaden Kinderklinik DKD, Wilhelmshaven Kinderarztpraxis, Wilhelmshaven Klinikum Kinderklinik, Winnenden Rems-Murr Kinderklinik, Witten Kinderarztpraxis, Wittenberg Innere Medizin, Worms Kinderklinik, Wuppertal Universitäts-Kinderklinik, Zams Kinderklinik, Zwettl Landesklinikum Gmünd-Waidhofen Kinderklinik


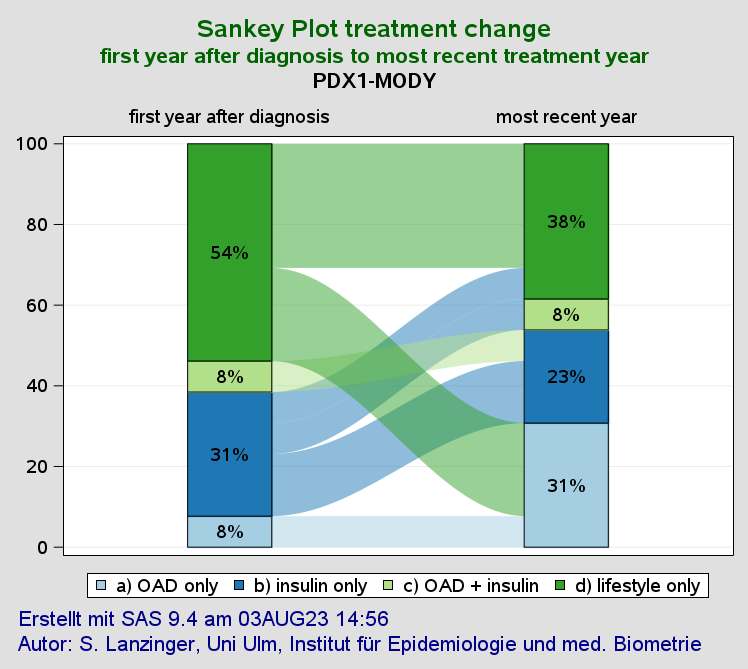

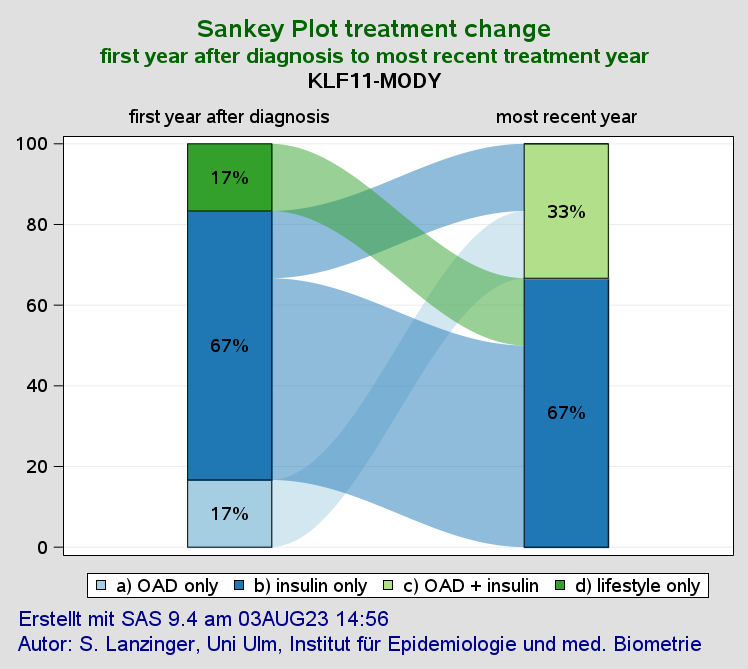


**B**

**A**

**C**


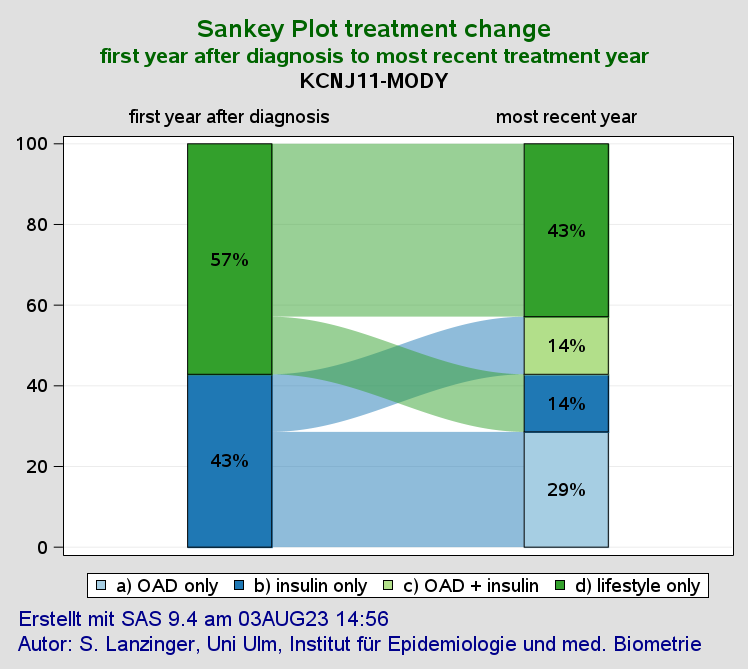


**C**

**Supplemental Figure 1** Sankey Plots showing changes in treatment from first year after diagnosis to the most recent year in
A) PDX1-, B) KLF11- and C) KCNJ11-MODY.
